# Supplementary material for: The Positive Association between Muscle Mass and Bone Status Is Conserved in Men with Diabetes: A Retrospective Cross-Sectional and Longitudinal Study
Source: J Clin Med. 2022 Sep 13;11(18):5370. doi: 10.3390/jcm11185370 (PMC9505062; doi:10.3390/jcm11185370)
Supplement: Supplementary file 1 [file jcm-11-05370-s001.zip › jcm-1835813-supplementary.pdf]

Supplementary Materials

Table S1. Time-dependent change of bone-related parameters, ASMI, and body profile data.

	**DM (n = 318)**			**Non-DM (n = 88)**		
**Variables**	**Initial Data**	**Follow-Up Data**	***p***	**Initial Data**	**Follow-Up Data**	***p***
Lumbar spine BMD (g/cm^2)	1.154±0.182	1.177±0.192	<0.001	1.041±0.195	1.060±0.189	0.011
Femoral neck BMD (g/cm^2)	0.969±0.151	0.972±0.155	0.052	0.932±0.170	0.946±0.164	0.010
Total hip BMD (g/cm^2)	1.044±0.160	1.049±0.164	0.002	0.956±0.162	0.973±0.154	0.001
TBS	1.451±0.099	1.468±0.096	<0.001	1.460±0.096	1.479±0.083	0.003
ASMI	7.778±1.009	7.826±1.039	0.250	7.419±0.931	7.519±1.156	0.001
Weight	74.848±12.259	75.032±12.204	0.717	69.988±11.250	71.338±11.336	<0.001
Height	169.706±6.089	169.589±6.221	0.002	172.119±6.944	172.222±7.130	0.040
BMI (kg/m^2)	25.935±3.689	26.030±3.636	0.261	23.591±3.369	23.998±3.193	0.001

DM: diabetes mellitus, ASMI: appendicular skeletal muscle index, BMD: bone mineral density, TBS: trabecular bone score, BMI: body mass index
